# Triplane technique for breast reconstruction after breast cancer surgery: A case series report

**DOI:** 10.1097/MD.0000000000037559

**Published:** 2024-03-29

**Authors:** Xiao-Juan Yang, Wen-Huan Wang, Jie-Ya Zou, Ji Wang, Zhuang-Qing Yang

**Affiliations:** a The Department of Breast Surgery, The Third Affiliated Hospital of Kunming Medical University, Yunnan Provincial Cancer Hospital, Kunming, Yunnan, P.R. China.

**Keywords:** breast cancer, breast-conserving surgery, prosthesis implantation, triplane technique

## Abstract

**Rationale:**

Implant-based breast reconstruction is an important method for post-mastectomy breast reconstruction. Currently, the most commonly used technique is the biplane technique. However, the high rate of postoperative complications, the inability of pockets to accommodate larger implants, and the expensive costs of biological mesh make the development of new surgical methods urgent. The triplane technique for breast reconstruction is an ideal candidate method.

**Patient concerns:**

The main local symptoms were breast lump, abnormal breast skin, nipple discharge, and abnormal nipple or areola in 24 patients.

**Diagnoses:**

The study included 24 female patients who underwent breast reconstruction using the triplane technique after radical breast cancer surgery.

**Interventions:**

The surgical procedure involved measuring the dimensions of the breast, designing the incision, and creating a pocket for the implant using the triplane technique, which includes the pectoralis major muscle, the pectoralis major fascia continuing to the rectus abdominis fascia, and the latissimus dorsa muscle fascia continuing to the rectus abdominis fascia. Postoperative follow-up included regular assessments of pain and evaluation of breast appearance.

**Outcomes:**

No cases of postoperative infection were observed in all patients. During the 1-year follow-up period after surgery, 5 patients (20.8%) who needed radiotherapy after mastectomy for cancer showed slight darkening of skin flap pigment after using the triplane technique implant. No cases of exposure or infection of the expanders were reported, and 1 patient underwent expander replacement with a permanent prosthesis. All patients expressed satisfaction with the reconstructed breast shape. The 10 patients (41.7%) experiencing postoperative swelling and pain. However, the pain gradually subsided during the postoperative recovery period. No cases of local recurrence or distant metastasis of breast cancer were observed during the 1-year-follow-up period.

**Lessons:**

The triplane technique for breast reconstruction after breast cancer surgery provides good implant coverage, reduces the risk of complications, and is cost-effective.

## 1. Introduction

Breast cancer is the most common malignant tumor in women, and its incidence is increasing steadily, with a trend toward younger ages. Studies have also confirmed that the age at onset of breast cancer in East Asian countries, including China, is younger compared to Western countries, with a median age of onset of 45 to 49 years.^[1]^ The loss of breast shape after breast cancer surgery has a negative impact on the psychological, physiological, and quality of life of female patients.^[2]^ Additionally, with the improvement in early screening and treatment of breast cancer, the survival rate of patients has significantly increased. As a result, more breast cancer patients are requesting breast preservation to enhance their quality of life and reduce psychological distress caused by breast loss. Although most patients prefer breast preservation in their surgical treatment, breast reconstruction serves as an alternative option for patients who are unable or unwilling to undergo breast preservation. Implant-based breast reconstruction is an important method for post-mastectomy breast reconstruction. In the United States, the number of patients undergoing breast augmentation or reconstruction using implants increased by 44% in 2021 compared to 2020.^[3]^ The choice of implant surface coverage technique in breast reconstruction after breast cancer surgery affects the aesthetic outcomes, complications, and cost-effectiveness. Currently, the most commonly used technique is the biplane technique. However, the biplane technique has limitations regarding lower implant coverage and is subject to certain restrictions in clinical use. To address these limitations, the triplane technique has been proposed. The triplane technique involves creating a pocket for the implant using the pectoralis major muscle, the pectoralis major fascia continuing to the rectus abdominis fascia, and the latissimus dorsi muscle fascia continuing to the rectus abdominis fascia. This technique not only eliminates the need for expensive biological mesh but also provides a larger and more complete pocket for accommodating larger implants, reducing the occurrence of postoperative complications.

## 2. Cases summary

From January 2020 to December 2022, 24 breast cancer patients underwent implant-based breast reconstruction using the triplane technique after radical mastectomy at the Department III of Breast Surgery, The Third Affiliated Hospital of Kunming Medical University, Yunnan Provincial Cancer Hospital. The patients’ ages ranged from 27 to 58 years, with an average age of 43 years. All patients were female. Tumor sizes ranged from 2.0 to 4.0 cm, with an average size of 2.9 cm. Among the cases, 14 tumors were located in the left breast and 10 tumors were located in the right breast. Specifically, 10 tumors were in the central area, 4 tumors were in the upper quadrant adjacent to the nipple, 6 tumors were in the upper outer quadrant adjacent to the nipple, and 4 tumors were in the lower outer quadrant adjacent to the nipple. The pathologic types included 4 cases of ductal carcinoma in situ and 20 cases of invasive carcinoma. Among the invasive carcinomas, 3 cases were classified as Luminal A subtype, 12 cases as Luminal B subtype, 3 cases as human epidermal growth factor receptor 2-positive subtype, and 2 cases as triple-negative subtype (Table 1).

**Table 1:** Clinicopathological features.

	N(%)
Number of patients	24
Position	
Left breast	14 (58.3)
Right breast	10 (41.7)
Age (yr)	
Age range	27–58
Mean age	43
Pathological type	
Ductal carcinoma in situ	4 (16.7)
Invasive carcinoma	20 (83.3)
T-stage	
Tis	4 (16.7)
T1	9 (37.5)
T2	11 (45.8)
N-stage	
N0	19 (79.2)
N1	5 (20.8)
Molecular typing of breast cancer	
Luminal A	3 (15.0)
Luminal B	12 (60.0)
HER2 positive type	3 (15.0)
Triple-negative	2 (10.0)

HER2 = human epidermal growth factor receptor 2.

## 3. Operative methods

**Figure 1.**
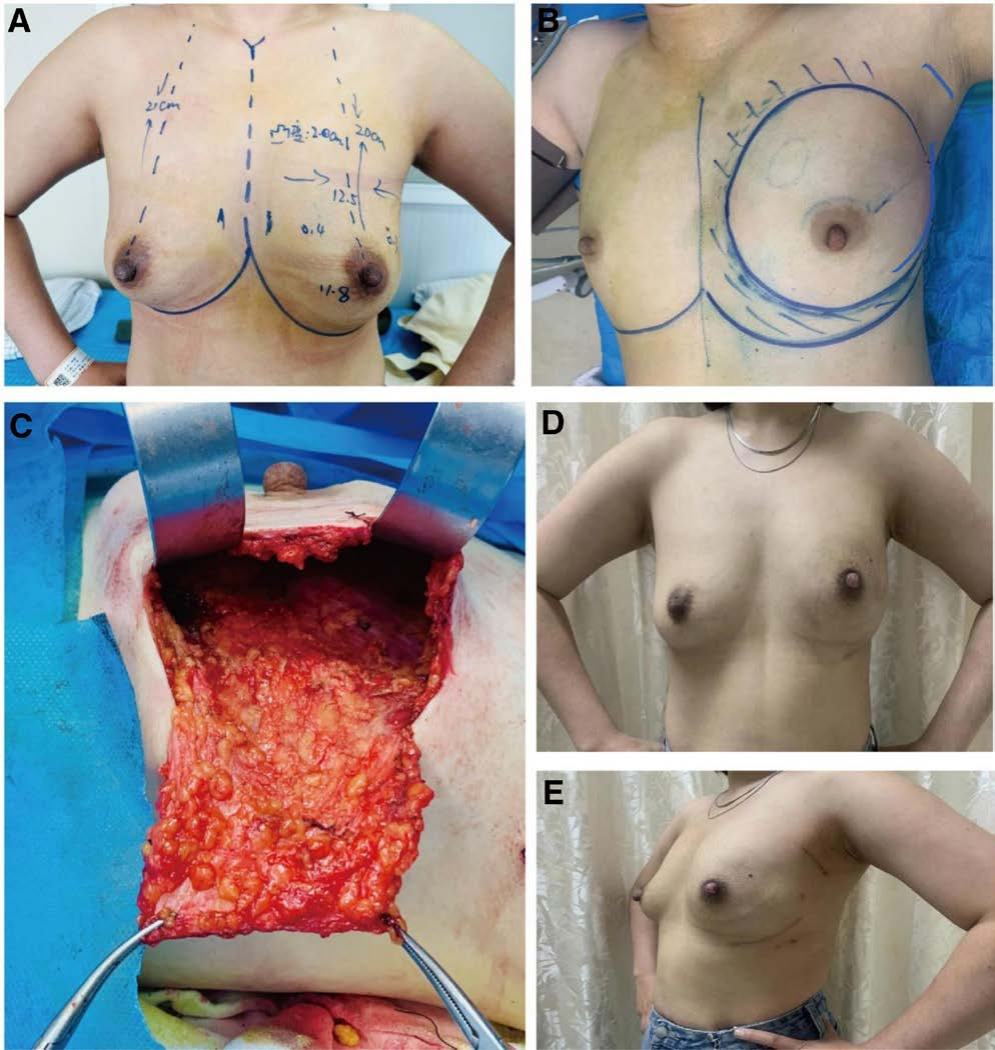
Demonstration of triplane technique. (A) Preoperative breast measurement, (B) Surgical design, (C) Complete surgical dissection of triplane tissue, (D,E) 3 mo after surgery.

Surgical Procedure Breast Measurements: The width, height, convexity, and subcutaneous fat thickness of the affected breast were measured to preliminarily determine the size of the implant (Fig. 1A).

Steps and Methods: Incision Design: A spindle-shaped incision was made at the site of the puncture needle, following the external fold of the breast wall and a 6 cm arc-shaped incision at the axilla (Fig. 1B). Surgical Steps: The patient was placed in a supine position with the affected arm abducted at approximately 80°. Methylene blue was injected subcutaneously near the areola, followed by routine disinfection, draping, and hand wrapping. A 3 cm incision was made in the axilla for sentinel lymph node biopsy. If lymph node metastasis was found, axillary lymph node dissection was performed. Another incision, approximately 6 cm in length, was made along the external fold of the affected breast. The skin, subcutaneous tissue, and deep and superficial fascia were sequentially dissected, and the glandular tissue was completely removed, with the weight of the tissue recorded. Multiple specimens were taken from below the nipple and areola for rapid frozen section examination to determine the presence of infiltrating cancer cells. If infiltration was observed, nipple-sparing was abandoned, and a spindle-shaped incision that included the nipple-areola complex was selected. To prevent postoperative ischemic necrosis of the nipple-areola complex, careful separation without excessive thinning was performed, avoiding the use of electrocautery. The thoracodorsal fascia was dissected from its anterior border, preserving the integrity of the serratus anterior fascia and pectoralis major fascia (Fig. 1C), with attention given to protect the feeding vessels to ensure blood supply to the fascial flap. A pocket for the implant was created, fully releasing the space between the major and minor pectoral muscles, extending medially to the parasternal line, superiorly to the second intercostal space, and inferiorly 1 to 2 cm below the inframammary fold, with care taken to preserve the thoracic nerve to avoid long-term atrophy of the pectoralis major muscle. Depending on the degree of breast ptosis, the distal end of the pectoralis major muscle was detached if severe ptosis was present. The wound was thoroughly rinsed with normal saline or sterile water, hemostasis was ensured, and a drainage tube was placed. Following the preoperative breast measurements and resection weight of the breast tissue, an appropriate implant or tissue expander was placed. The free edge of the intact fascial adipose tissue flap was sutured to the lateral chest wall, completely wrapping the implant. The subcutaneous and skin layers were sutured layer by layer, and sterile dressings were applied. Postoperative wound healing and breast appearance were monitored (Fig. 1D and E).

Postoperative: Regular follow-up evaluations were conducted to assess postoperative pain and breast appearance. Comprehensive examinations were performed every 3 months to detect any local recurrence or distant metastasis of the tumor. Among the 24 patients, 5 underwent tissue expander implantation used the triplane technique after radical mastectomy for breast cancer, and 19 patients underwent direct implantation of breast implants using the triplane technique after radical mastectomy. The sizes of the implanted implants ranged from 235cc to 300cc, with an average size of 256.6cc (Table 2).

**Table 2:** The size of the implant.

The size of the implant (cc)	N(%)
235	9 (47.4)
270	8 (42.1)
300	2 (10.5)

## 4. Safety, satisfaction, and oncology outcomes

Postoperative follow-up ranged from 3 to 27 months. Among all the patients, 15 patients had minor complications, accounting for 62.5%. The most common complication was postoperative swelling and pain, which were reported in 10 patients (41.7%). However, these symptoms gradually disappeared during the postoperative recovery period. The skin flap color was slightly deepened after radiotherapy in 5 (20.8%) patients receiving radiotherapy after radical mastectomy with 3-plane technique dilator implantation and 1 patient has already undergone expander replacement with implant surgery. No cases of postoperative infection, seroma, prosthesis displacement, hemorrhage, artificial exposure, capsular contracture or congestion of skin flap were observed in all patients. All patients were satisfied with the reconstructed breast appearance. The 19 patients (79.2%) were more satisfied and 5 (20.8%) were satisfied. During the 1 year of follow-up, no cases of local recurrence or distant metastases of breast cancer were observed, and none of the patients died (Table 3).

**Table 3:** Surgical safety and the oncology outcome of the operation.

Characteristic	N (%)
Postoperative complications	
Yes	15 (62.5)
No	9 (37.5)
Types of postoperative complications	
Postoperative swelling and pain	10 (41.7)
Pigmentation of skin flap	5 (20.8)
Postoperative infection	0
Seroma	0
Prosthesis displacement	0
Hemorrhage	0
Artificial exposure	0
Capsular contracture	0
Congestion of skin flap	0
Patient satisfaction	
More satisfied	19 (79.2)
satisfaction	5 (20.8)
dissatisfy	0
Tumor recurrence	
Yes	0
No	24
Tumor metastasis	
Yes	0
No	24
Patient death	
Yes	0
No	24

## 5. Discussion

Breast cancer is the most common malignancy in women worldwide. According to the Global Cancer Observatory 2020,^[4]^ which released data on cancer incidence, mortality, and trends in 185 countries/regions, the incidence of breast cancer has been increasing year by year and trending toward a younger age group. The comprehensive treatment methods for breast cancer include surgery, chemotherapy, endocrine therapy, targeted therapy, and radiation therapy. Among them, surgery is an important treatment modality. With the continuous updates and optimization of breast cancer treatments, the survival time of early-stage breast cancer patients has been prolonged, and the demand for breast aesthetics has also been increasing, thereby improving the quality of life. For patients who cannot or are unwilling to undergo breast-conserving surgery, breast reconstruction has become a preferred choice for more patients, leading to an increasing number of post-mastectomy breast reconstructions.^[5]^

Breast reconstruction methods include autologous breast reconstruction and implant-based breast reconstruction.^[6]^ Implant-based breast reconstruction has advantages such as shorter operative time, faster patient recovery and elimination of donor site morbidity compared to autologous breast reconstruction.^[7]^ The planes for implant placement in post-mastectomy breast reconstruction include the pre-pectoral plane (between the skin flap and the pectoralis major muscle) and the sub-pectoral plane (between the pectoralis major muscle and the chest wall).^[8]^ The choice of implant placement plane results in different outcomes and complications. Complications of implant reconstruction include infection, capsule contracture, pain syndrome, implant rupture, and implant edge sensation.^[9]^ Historically, the pre-pectoral plane has been associated with higher complications. However, with the improvement of surgical techniques and the use of acellular dermal matrix, these complications have significantly decreased, leading to the resurgence of pre-pectoral implant.^[7,10]^ Tebbetts proposed the concept of dual-plane breast augmentation in 2001,^[11]^ which solved the problem of implant displacement and morphological changes caused by pectoral muscle contraction. However, for patients undergoing radical surgery for breast cancer, due to the complete removal of glandular tissue, there is insufficient soft tissue coverage in the lower outer pole of the implant, which is also one of the clinical challenges for breast reconstruction in breast cancer patients. The rapid development of implant materials has significantly reduced postoperative complications and improved the aesthetic outcomes of breast reconstruction when applying artificial materials to effectively cover the implant. However, due to the high cost of acellular dermal matrix/TiLoop mesh, most patients are unable to afford such expensive materials, which limits the clinical application of this technique.

At the Breast Center of Yunnan Cancer Hospital, 24 patients with breast cancer underwent radical surgery with triplane technique and breast implantation for breast reconstruction. All patients expressed satisfaction with the postoperative breast shape, and there was no increase in operation time, length of hospital stay, or the use of postoperative drainage due to the introduction of this new technique. The implanted implant sizes ranged from 235 to 300 cc for patients in this group, none of whom experienced complications such as implant exposure or infection due to incomplete tissue coverage of the implant surface. The most common complication observed in patients was postoperative swelling and pain, which gradually disappeared as the recovery progressed. No cases of tumor recurrence, metastasis, or death were observed during the short-term follow-up period. However, there are some limitations in this report, the number of patients in this report is limited, and the results of the 3-plane technique can only be provided to a certain extent. Since the 3-plane technique has just been used, not enough patients in our hospital have chosen the 3-plane technique for breast reconstruction, and it is not enough to conduct statistically significant analysis with the traditional 2-plane breast reconstruction. It is hoped that sufficient number of clinical cases can be collected in the follow-up clinical observation to provide results in this respect. In addition, the effect of breast reconstruction needs to be observed for a longer period of time, and we will continue to follow-up these patients to extend the observation time. The triplane technique for breast reconstruction after breast cancer surgery provides good implant coverage, reduces the risk of complications, and is cost-effective.

## Acknowledgments

The authors are very grateful to the patients for providing the data.

## Author contributions

**Data curation:** Xiao-Juan Yang, Wen-Huan Wang, Jie-Ya Zou, Ji Wang.

**Writing – original draft:** Xiao-Juan Yang.

**Writing – review & editing:** Xiao-Juan Yang, Zhuang-Qing Yang.
